# Online hemodiafilteration use in children: a single center experience with a twist

**DOI:** 10.1186/s12882-020-01957-9

**Published:** 2020-07-28

**Authors:** Magid A. A. Ibrahim, Ihab Z. ElHakim, Dina Soliman, Muhammad A. Mubarak, Ragia M. Said

**Affiliations:** 1 Department of Pediatrics, Faculty of Medicine, Ain Shams University, Cairo, Egypt; 2 Department of Clinical Pathology & Immunology, Faculty of Medicine, Ain Shams University, Cairo, Egypt

**Keywords:** ESRD, HD, OL-HDF, Kt/V, Children

## Abstract

**Background:**

Haemodiafilteration (HDF) is a promising new modality of renal replacement therapy (RRT). It is an improvement in the quality of hemodialysis (HD) and thus in the quality of patients’lives. The main obstacle to using HDF is the cost, especially in developing countries. The purpose of this study was to evaluate the benefits of incorporating HDF with different regimens in the treatment of children with end stage renal disease (ESRD).

**Methods:**

Thirty-four children with ESRD on regular HD in Pediatric Dialysis Unit, Children’s Hospital, Ain Shams University were followed up in 2 phases: initial phase (all patients: HD thrice weekly for 3 months) and second phase, patients were randomized into 2 groups, HDF group and HD group, the former was subdivided into once and twice weekly HDF subgroups. Evaluation using history, clinical and laboratory parameters at 0, 3, 9 and 18 months was carried out.

**Results:**

On short term, we found that the HDF group was significantly superior to HD group regarding all clinical and laboratory parameters. Also, twice HDF subgroup was significantly superior to once HDF subgroup. This was confirmed on long term follow up, but the once HDF proved comparable to twice subgroup.

**Conclusions:**

Incorporating online hemodiafilteration (OL-HDF) in the RRT of children was beneficial in most of the clinical and laboratory parameters measured. It’s not all or non; OL-HDF, even once a week, can improve outcomes of HD without significantly affecting the cost.

## Background

Hemodiafiltration (HDF) includes diffusive and convective solute removal by ultrafiltering 20% or more of the blood volume and maintaining fluid balance by infusing sterile replacement fluid into the patient’s blood. In online hemodiafiltration (OL-HDF), replacement fluid is obtained by online filtration of dialysate through a series of bacteria- and endotoxin-retaining filters [1].

HDF expands the spectrum of uremic toxin removal from small-sized solutes to middle-sized and large molecular weight solutes [2].

Conservation of residual renal function (RRF) in children with ESRD has many benefits including better growth and nutrition, anemia correction, calcium-phosphorous balance, better control of blood pressure and decreasing cardiovascular risk [3]. HDF, though not the best method for conserving RRF, is a better way of renal replacement therapy compared to conventional HD in the context of decreasing intradialytic events that have negative effects on RRF. OL-HDF was associated with a 30% reduction in all-cause mortality compared with conventional hemodialysis [4, 5].

The group of middle molecule, defined by a molecular weight ≥ 500 D, is mainly composed of small peptides [6]. Many of these are implied in cardiovascular disease, by causing inflammation, endothelial damage, smooth muscle cell proliferation, activation of coagulation or by interfering with calcium/phosphorus homeostasis [7, 8]. Many randomized controlled studies stated that OL-HDF showed greater efficiency in removing small solutes and in reducing basal levels of phosphate and parathyroid hormone, [9, 10] as well as reducing inflammatory parameters such as CRP and IL-6 and pro-inflammatory CD14^+^ CD16^+^ cells [11–14].

***Mazairac*et al.*, (2013)*** suggest that HDF, compared to HD, belongs to (higher cost, better health) corner [15]. However, ***McBrien and Manns (2013)*** proposed that HDF in fact belongs to (higher cost, no impact on health) area [16].

OL-HDF is not yet used worldwide in children and a small number of reports have been published on the effectiveness and safety of the procedure in children. As such, the clinical experience of OL-HDF is still limited, especially in the developing countries, including Egypt where the high cost of this new modality represents an important obstacle. Hence, our study was conducted to evaluate the benefits of implementing this modality with different regimens that can reduce the cost and provide a substantial benefit.

## Study design

### Subjects and sample collection

This study is a prospective comparative study (sequential clinical follow up study) and was conducted on children and adolescents following up in the Pediatric Dialysis Unit, Children’s Hospital, Ain Shams University. The study included a total of 34 children, 21 (61.8%) boys and 13 (38.2%) girls, their mean age was 14.7 **±** 3.5 years, they all had ESRD and were on regular hemodialysis, only 31 completed the study to its end. They fulfilled these ***Inclusion Criteria*:**1-On regular hemodialysis for at least 3 months.2-Can achieve actual extracorporeal blood flow rate (Q_b_) of at least 250 ml per min. ***Exclusion Criteria included:***1-Presence of any underlying rheumatological diseases or conditions that can increase inflammatory markers.2-Receiving immunosuppressive treatment or steroids.

This study lasted for 18 months divided into two phases:

I. **Initial phase (3 months):** All patients were evaluated by clinical and laboratory parameters initially, kept on conventional HD for 3 months, followed up and evaluated again after completing these 3 months ***(3 months Evaluation).***
II. **Second phase (15 months):** At the end of the initial phase, patients were randomized into 2 groups and 2 subgroups as follows:
  A. ***HDF group: were sub divided into 2 subgroups***
    i. ***Once weekly HDF subgroup (once subgroup):*** 10 patients were put on OL-HDF once weekly (middle dialysis session of the week, the remaining two sessions were conventional HD sessions) using dialysis machines available in the pediatric dialysis unit. One patient was lost from this group at 18 months evaluation because of transplantation.
    ii. ***Twice weekly HDF subgroup (twice subgroup):*** 12 patients were put on OL-HDF twice weekly (first and last dialysis sessions of the week, the remaining 3rd session was conventional HD). Two patients were lost from this group at 18 months evaluation because of referral to an adult HD unit.
  B. ***HD group:*** 12 patients were put on conventional hemodialysis thrice weekly to serve as controls for previous groups; being age and sex matched with them.

**After this randomization, they were evaluated again twice as follows:**

- *Evaluation at 9 months:* after 9 months from the beginning of the study to assess short term effects of HDF ***(9 months evaluation).***
- *Evaluation at 18 months: after* 18 months from the beginning of the study to assess long term effects of HDF ***(18 months evaluation).***

We used the percentage change in the measured parameters to assess the changes that occurred in these parameters’ values during the study:

Percent change = ((value after-value before)/ value before)*100 [17].

To assess the short term changes we used the percentage change between ***3 months evaluation*** and ***9 months evaluation*** (Change 3–9) and to assess the long term changes we used the percentage change between ***3 months evaluation*** and ***18 months evaluation*** (Change 3–18).

### Methods

An informed consent was taken from every patient or his/ her caregiver.

All patients were subjected to.

***History taking &clinical examination including:*** duration on HD in years, blood flow rate (Q_b_), dialysate flow rate (Q_d_), dialyzer size, dialysis related complications: the intradialytic symptomatic hypotension (ISH) and post dialysis fatigue were systematically recorded and expressed as frequency/ month. Drug history especially total Erythropoietin (Epo) dose/ month, Epo dose/kg, and Epo/Hct ratio. C**linical examination with** stress on Anthropometric measures: Weight (Wt) and Height (Ht), both are expressed as Standard Deviation Score ***(SDS)*** [18] Blood pressure (BP): Predialysis systolic and diastolic BP were measured using electronic blood pressure monitors in our unit and mercury sphygmomanometers at home and recorded at each evaluation and BP percentiles were determined [19].

#### The laboratory parameters evaluation

A morning predialysis two venous blood samples (2 mL each) were obtained from each participant. The first sample was used for measurement of calcium, phosphorus, parathyroid hormone (PTH). β_2_ microglobulin (β_2_m), interleukin 6 (IL-6) and hs-CRP by ELISA technique. The second sample was used for performing complete blood picture (CBC). Hemoglobin (Hb) and Hematocrit (Hct) and were evaluated in accordance to age and sex [20] to assess severity and progress of anemia.

**Assessment of adequacy of dialysis (Kt/V):** using online conductivity monitoring (OCM) by dialysis machine. It was calculated as the sum Kt/V of the 3 dialysis sessions of the week and expressed as Kt/V/week.

**The costs versus benefit calculation:**

- **The costs**: we calculated only the current costs in 28 days period at each evaluation and expressed as US $/month. They were expressed as follows:
  **Dialysis costs:** It included the sum of all dialysis sessions costs per 28 days, it included prices of dialyzers, blood lines, substitution fluid lines and diasafe filters.
  **Non-dialysis costs:** It included the sum of all non-dialysis costs that were feasible to be recorded. It included prices of Erythropoietin Simulating Agents (ESA), Iron therapy, activated vitamin D and phosphate binders.
  **Net costs:** It is the sum of the previous two, dialysis costs plus non-dialysis costs.
- **The benefits:** we created a **“benefit score”** in our study depending on the long-term improvement, no change or deterioration in clinical and laboratory parameters. We determined a cut off value of 25% percent change between 3 months and 18 months evaluations to signify improvement or deterioration. Then we calculated the **“Total benefit score”** for each patient which is the algebraic sum of benefit score for each of the 8 parameters (ISH frequency, Post-dialysis fatigue frequency, Hemoglobin, Calcium, Phosphorus, PTH, β_2_m, IL-6) in each patient.
- **The benefit Category:** according to the change of the total benefit score, we categorized our patients into 3 categories:

- Benefit: positive total benefit score.
- No change: total benefit score equals zero.
- Deterioration: negative total benefit score.

d) **The cost / benefit ratio:** In each patient we calculated the incremental costs (the net costs at 18 months evaluation - the net costs at 3 months evaluation) then we divided it by the total benefit score mentioned above and expressed as EGP/benefit (recalculated as US$). This ratio was used to compare between different patients’ groups and subgroups to decide which is more cost effective.

*Note: when the total benefit score of a patient is negative or equals zero (as in some patients in HD group), this patient was cancelled from statistics of cost benefit ratio.*

#### Dialysis methodology

OL-HDF was performed using the Fresenius dialysis system (Fresenius Medical Care, Bad Homburg, Germany). The same dialyzers’ configurations, the same surface area of the dialyzers using polysulfone membrane-based dialyzer during OL-HDF and the same blood flow rate and dialysate flow rate (500 mL/min) and temperature (36 °C) were used during both conventional HD and OL-HDF. Bicarbonate powder cartridges (Fresenius Medical Care) were used with ultrapure water for the preparation of bicarbonate-containing dialysis fluid. The substitution fluid was prepared from the dialysis fluid by one additional step of controlled ultrafiltration, before it was infused post-filter into the blood (post dilution mode). We used the on-line system (Fresenius Medical Care, Bad Homburg, Germany), which is integrated into the dialysis machine and consists of two ultrafilters, an infusate pump module and disposable infusate lines. The infusate was prepared continuously by double-stage ultrafiltration. Both filters were subjected to automated membrane integrity tests before dialysis and were replaced after 100 treatments or 12 weeks of use, whichever comes first. The on-line HDF was performed with an infusion rate of one fifth to quarter the blood flow rate guided by trans-membrane pressure (TMP) maintained below 200.

#### Diet

No change in “diet routine” and no “special meal plans” were involved in our study.

## Statistical methods

The results were tabulated, graphically represented and analyzed. A *p*-value of < 0.05 was considered significant. Standard computer program SPSS (**S**tatistical **P**ackage for **S**ocial **S**ciences) for Windows, release 13.0 (SPSS Inc., USA) was used for data entry and analysis [21].

## Results

**Fig. 1:**
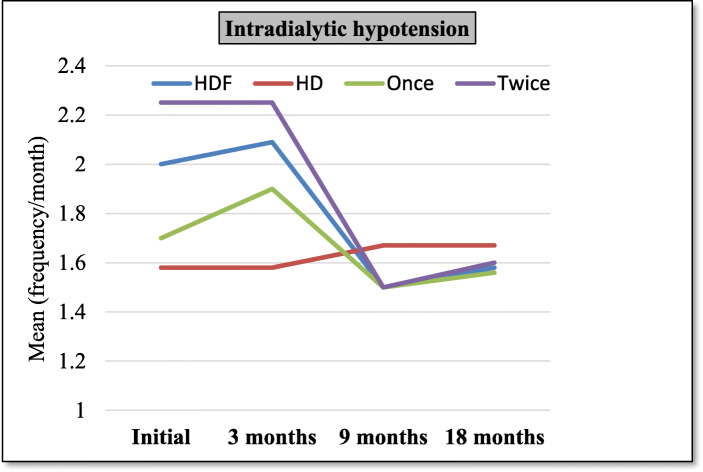
Line chart for intradialytic hypotension frequency means of different groups

**Fig. 2:**
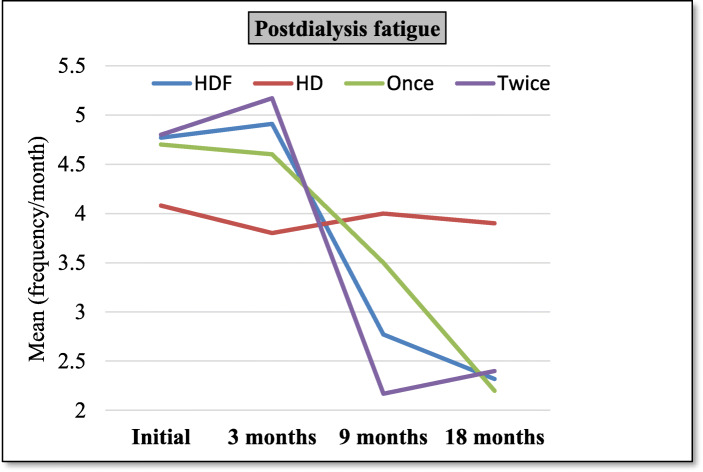
Line chart for post dialysis fatigue frequency means of different groups

-Descriptive statistics and comparisons between the initial and 3 months evaluations (in all patients as one group) as regards **clinical parameters, laboratory parameters** and **drugs & costs** revealed that the weight SDS was significantly higher at 3 months evaluation than initial one (*p* < 0.05) and the Epo dose/kg was significantly lower at 3 months evaluation than initial one (*p* < 0.05), no other significant difference was observed between the two evaluations in other parameters,

- Descriptive statistics and comparisons of HDF group versus HD group and once subgroup versus twice subgroup, at 3 months evaluation, 9 months evaluation, and 18 months evaluation are shown in Tables 1, 2, 3 & Figs. 1, 2. Height SDS was significantly higher in HDF group compared to HD at 3, 9 and 18 months. β2m and IL-6 were significantly lower in HDF group and in twice HDF group at 18 months while hs-CRP was significantly lower in HDF group at 9 and 18 months. Kt/V/week was significantly higher in HDF group and in twice HDF group at 9 and 18 months.

**Table 1:** Comparison between different patients’ groups as regards clinical parameters at 3, 9 & 18 months’ evaluation

		3 months			9 months			18 months		
Variable	Group	Mean	±SD	p	Mean	±SD	p	Mean	±SD	p
**Weight** (SDS)	HDF	−4.90	4.26	0.1	−5.00	3.29	0.1	−4.40	3.11	0.1
	HD	− 7.13	3.86		−6.92	3.92		−6.60	4.00	
	Once	−4.54	3.15	0.7	−4.13	2.97	0.2	−3.64	2.73	0.3
	Twice	−5.23	5.22		−5.78	3.53		− 5.09	3.41	
**Height** (SDS)	HDF	−4.09	2.4	**0.00**	− 4.02	2.33	**0.00**	−3.57	2.46	**0.00**
	HD	−6.07	1.64		−6.03	1.67		−5.87	1.73	
	Once	−3.57	2.05	0.4	−3.63	1.79	0.5	−3.04	1.99	0.3
	Twice	−4.55	2.71		−4.37	2.78		−4.04	2.84	
**Intradialytic hypotension** (Freq/month)	HDF	2.09	0.92	0.1	1.5	0.51	0.4	1.58	0.60	0.7
	HD	1.58	0.66		1.67	0.65		1.67	0.65	
	Once	1.9	0.87	0.4	1.5	0.5	1	1.56	0.5	1
	Twice	2.25	0.96		1.5	0.5		1.6	0.69	
**Postdialysis fatigue** (Freq/month)	HDF	4.91	0.86	**0.00**	2.77	0.06	**0.01**	2.32	0.67	**0.00**
	HD	3.8	0.93		4	1.27		3.9	0.79	
	Once	4.6	0.96	0.1	3.5	0.97	**0.00**	2.2	0.4	0.6
	Twice	5.17	0.71		2.17	0.7		2.4	0.84	

*SDS* Standard Deviation Score

**Table 2:** Comparison between different patients’ groups as regards laboratory parameters at 3, 9 & 18 months evaluation

		3 months			9 months			18 months		
Variable	Group	Mean	±SD	p	Mean	±SD	p	Mean	±SD	p
**Hb** (gm/dl)	HDF	10.15	1.57	0.2	10.95	1.61	0.6	11.7	1.96	0.2
	HD	10.9	1.9		10.6	1.7		10.9	1.4	
	Once	10.5	1.7	0.2	10.8	1.8	0.7	11.8	2.3	0.7
	Twice	9.7	1.3		11.05	1.4		11.5	1.7	
**Ca** (mg/dl)	HDF	7.88	1.30	0.7	8.85	1.55	0.4	9.24	2.21	0.5
	HD	8.13	2.1		8.3	2.28		8.7	1.8	
	Once	8.07	1.04	0.5	9	1.5	0.6	9.7	1.8	0.1
	Twice	7.73	1.5		8.7	1.64		8.8	2.5	
**P** (mg/dl)	HDF	7.07	2.65	**0.04**	6.55	1.94	0.6	6.73	1.95	0.4
	HD	5.55	1.3		6.87	1.59		6.1	2.6	
	Once	8.4	3.2	**0.01**	7.4	1.45	0.05	7.4	2	0.1
	Twice	5.9	1.46		5.8	2.05		6.1	1.7	
**PTH** (pg/ml)	HDF	444.91	323	0.1	366.5	328.8	0.3	193.6	205	0.8
	HD	322	392		319	384.6		243	346.6	
	Once	429	291.2	0.8	396.9	277.1	0.3	217.6	240.9	0.5
	Twice	457	361		341.2	376.9		172	178	
**β**_**2**_**m** (μg/ml)	HDF	8.2	1.77	0.5	6.48	2.03	0.09	5.05	1.77	**0.00**
	HD	8.7	3.2		7.9	2.39		7.5	1.8	
	Once	8.8	1.59	0.1	7.35	2.02	0.06	5.9	1.9	**0.03**
	Twice	7.67	1.78		5.75	1.8		4.2	1.2	
**IL-6** (pg/ml)	HDF	237.5	96.59	0.6	192.05	83.5	0.07	119.74	56.26	**0.00**
	HD	222.9	90.1		245.8	76.74		250	92.9	
	Once	232.5	76.4	0.8	202.5	65.03	0.6	147	49	**0.03**
	Twice	241.6	113.9		183.3	98.5		95	52.4	
**hs-CRP** (μg/ml)	HDF	14.41	8.34	0.8	8.5	6.06	**0.03**	4.58	5.18	**0.00**
	HD	14.8	9.2		14.9	8.86		14.75	9.5	
	Once	12.8	9.7	0.4	9.7	7.37	0.6	6.1	7	0.2
	Twice	15.7	7.1		7.5	4.8		3.15	2.1	

*Hb* Hemoglobin, *Ca* Calcium, *P* Phosphorus, *PTH* Parathyroid hormone, *β*_*2*_*m* beta 2 microglobulin, *IL-6* interleukin-6, *hs-CRP* highly sensitive C-reactive protein

**Table 3:** Comparison between different patients’ groups as regards drugs and costs at 3, 9 & 18 months’ evaluation

		3 months			9 months			18 months		
Variable	Group	Mean	±SD	p	Mean	±SD	p	Mean	±SD	p
**Kt/V/wk**	HDF	4.24	0.11	0.9	4.89	0.48	**0.00**	4.93	0.41	**0.00**
	HD	4.25	0.13		4.2	0.1		4.3	0.12	
	Once	4.2	0.1	0.2	4.4	0.25	**0.00**	4.6	0.2	**0.00**
	Twice	4.2	0.12		5.26	0.24		5.3	0.3	
**Epo dose/kg** (U/kg)	HDF	201.9	51.13	0.2	194.6	48.7	0.3	183.8	50.20	0.8
	HD	180.3	53.5		176.3	52.2		181.4	38.8	
	Once	213.5	63.3	0.3	205.4	58.8	0.3	197.9	58.9	0.2
	Twice	192.2	38.4		185.6	38.7		171	39.6	
**(EPO/kg) /Hct** **Ratio**	HDF	6.6	2.15	0.4	6.32	1.97	0.9	5.35	2.05	0.2
	HD	5.9	2.6		5.8	2.5		5.6	1.3	
	Once	6.8	2.4	0.3	6.5	2.6	0.2	5.8	2.5	0.5
	Twice	6.4	1.57		5.5	1.39		4.9	1.45	
**Iron dose** (mg/month)	HDF	183	77.97	0.2	157.6	44.27	0.5	135.26	36.87	0.2
	HD	165	82.4		151.6	44		145	34.2	
	Once	198	110	0.7	151	33.8	0.5	140	46.6	0.8
	Twice	170.8	36		162.5	40		131	27.2	
**Dialysis costs** (EGP/month) (US$/month)	HDF	1570 (209.33)	4.6 (0.613)	0.1	2247 (299.6)	224 (29.867)	**0.00**	2239 (298.533)	225.8 (30.107)	**0.00**
	HD	1567 (208.93)	3.34 (0.445)		1568 (209.067)	3.25 (0.433)		1568 (209.067)	3.25 (0.433)	
	Once	1569 (209.2)	21.52 (2.869)	0.4	2007 (267.6)	2.63 (0.35)	**0.00**	2007 (267.6)	2.63 (0.35)	**0.00**
	Twice	1571 (209.47)	4.74 (0.632)		2447 (326.267)	2.63 (0.35)		2448 (326.4)	3.49 (0.465)	
**Non-Dialysis costs** (EGP/month) (US$/month)	HDF	1212 (161.6)	184.9 (24.653)	**0.00**	1142 (152.267)	183.2 (24.427)	0.07	1103 (147.067)	217 (28.933)	0.6
	HD	841.7 (112.227)	407.9 (54.387)		839 (111.867)	408 (54.4)		1012 (134.933)	194 (25.867)	
	Once	1162 (154.933)	213.9 (28.52)	0.3	1110 (148)	209 (27.867)	0.6	1086 (144.8)	243 (32.4)	0.7
	Twice	1267 (168.933)	152 (20.267)		1183 (157.733)	155.7 (20.76)		1119 (149.2)	204 (27.2)	
**Net costs/m** (EGP/month) (US$/month)	HDF	2782 (370.933)	186.6 (24.88)	**0.00**	3389 (451.867)	302.3 (40.307)	**0.00**	3343 (445.733)	325 (43.333)	**0.00**
	HD	2409 (321.2)	408.7 (54.493)		2407 (320.933)	409 (54.533)		2580 (344)	196 (26.133)	
	Once	2732 (364.267)	216 (28.8)	0.3	3118.4 (415.787)	210 (28)	**0.00**	3094 (412.533)	243 (32.4)	**0.00**
	Twice	2839 (378.533)	154 20.533)		3631 (484.133)	157.2 (20.96)		3567 (475.6)	203.8 (27.173)	

*EPO* Erythropoietin, *Hct* hematocrit value

**Fig. 3:**
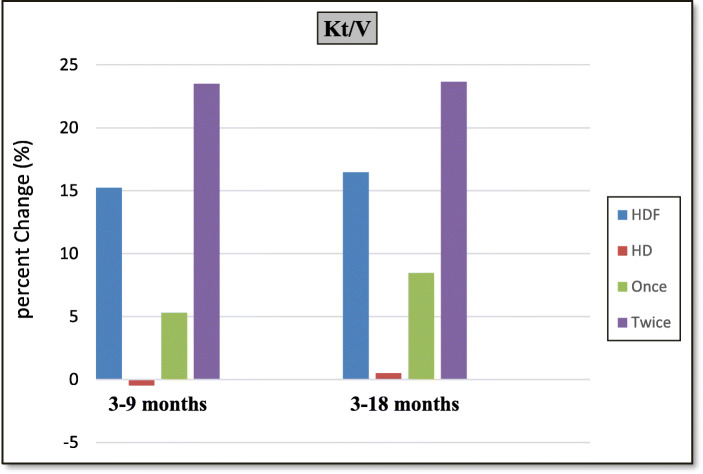
Column chart for percent changes in Kt/V in between different evaluations

**Fig. 4:**
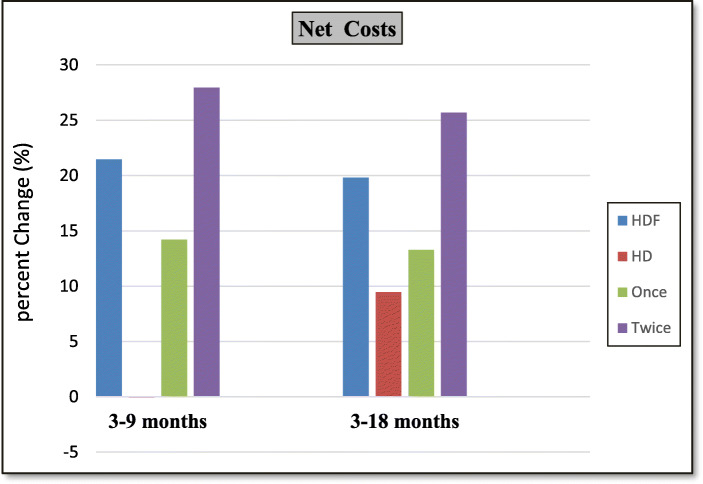
Column chart for percent changes in net costs in between different evaluations

-Percentage change between 3 months and 9 months evaluations (short term effects) and between 3 months and 18 months evaluations (long term effects) regarding different parameters are shown in Tables 4, 5, 6, Figs. 3, 4.

**Table 4:** Comparison between different groups as regards percentage change in clinical parameters in between 3 months & 9 months (short term) and 3 months & 18 months’ (long term) evaluations

		% change between 3 & 9 months (short term effects)			% change between 3 & 18 months (long term effects)		
Variable	Group	Mean	±SD	p	Mean	±SD	p
**Weight** (SDS)	HDF	−9.48	7.07	**0.01**	−23.10	13.50	**0.00**
	HD	−4.35	4.08		−10.77	10.01	
	Once	−10.19	7.69	0.6	−24.86	16.10	0.6
	Twice	−8.84	6.83		−21.52	11.33	
**Height** (SDS)	HDF	−10.15	17.03	**0.00**	−23.5	26.26	**0.00**
	HD	−0.87	2.21		−3.68	5.38	
	Once	−5.57	6.01	0.6	−20.38	17.86	0.5
	Twice	−13.97	22.12		−26.31	32.82	
**Intradialytic** **Hypotension** (Freq/month)	HDF	−19.69	33.97	0.1	−7.45	56.71	0.5
	HD	9.09	53.93		4.54	56.80	
	Once	−10.00	43.88	0.3	1.85	60.34	0.4
	Twice	−27.77	21.71		−15.83	55.06	
**Postdialysis** **fatigue** (Freq/month)	HDF	−42.95	19.98	**0.00**	−50.43	17.84	**0.00**
	HD	4.16	21.97		6.66	28.87	
	Once	−24.66	7.31	**0.00**	−48.51	19.22	0.8
	Twice	−58.19	12.70		−52.16	17.35

**Table 5:** Comparison between different groups as regards percentage change in laboratory parameters in between 3 months & 9 months (short term) and 3 months & 18 months’(long term) evaluations

		% change between 3 & 9 months (short term effects)			% change between 3 & 18 months (long term effects)		
Variable	Group	Mean	±SD	p	Mean	±SD	p
**Hb** (gm/dl)	HDF	8.76	13.53	**0.00**	18.70	17.90	**0.02**
	HD	−2.03	11.72		2.18	19.72	
	Once	2.70	11.09	**0.01**	16.36	21.74	0.6
	Twice	13.80	13.70		20.82	14.50	
**Ca** (mg/dl)	HDF	13.00	14.32	0.1	19.99	21.00	0.2
	HD	3.38	22.31		9.81	21.43	
	Once	11.95	14.67	0.7	21.85	14.09	0.7
	Twice	13.89	14.60		18.33	26.46	
**P** (mg/dl)	HDF	−3.05	24.13	**0.00**	−2.25	28.12	0.3
	HD	25.65	21.13		8.76	30.46	
	Once	−4.90	24.50	0.7	−10.69	23.51	0.2
	Twice	−1.51	24.80		5.33	30.91	
**PTH** (pg/ml)	HDF	−20.19	26.57	**0.04**	−51.97	32.97	**0.00**
	HD	−2.26	14.46		−10.56	29.65	
	Once	−7.69	13.23	**0.03**	−40.13	36.42	0.1
	Twice	−30.61	30.73		−62.63	27.0	
**β**_**2**_**m** (μg/ml)	HDF	−21.76	15.24	0.06	−38.75	15.63	**0.00**
	HD	−5.9	25.31		−9.55	24.87	
	Once	−17.28	14.66	0.2	−33.60	15.67	0.1
	Twice	−25.50	15.3		−43.39	14.82	
**IL-6** (pg/ml)	HDF	−19.54	14.05	**0.00**	−46.20	20.33	**0.00**
	HD	15.17	22.62		15.33	26.63	
	Once	−12.20	8.34	**0.01**	−32.07	13.96	**0.00**
	Twice	−25.66	15.17		−58.92	16.55	
**hs-CRP** (μg/ml)	HDF	−41.05	23.72	**0.00**	−66.93	19.60	**0.00**
	HD	9.33	40.23		2.94	28.47	
	Once	−23.16	16.00	**0.00**	−51.87	17.25	**0.00**
	Twice	−55.96	18.19		−80.48	8.54	

*Hb* Hemoglobin, *Ca* Calcium, *P* Phosphorus, *PTH* Parathyroid hormone, *β*_*2*_*m* beta 2 microglobulin, *IL-6* interleukin-6, *hs-CRP* highly sensitive C-reactive protein

**Table 6:** Comparison between different groups as regards percentage change in drugs and costs in between 3 months & 9 months (short term) and 3 months & 18 months’ (long term) evaluations

		% change between 3 & 9 months (short term effects)			% change between 3 & 18 months (long term effects)		
Variable	Group	Mean	±SD	p	Mean	±SD	p
**Kt/V/wk**	HDF	15.24	11.35	**0.00**	16.47	10.50	**0.00**
	HD	−0.46	4.83		0.51	5.13	
	Once	5.32	7.31	**0.00**	8.47	5.12	**0.00**
	Twice	23.50	6.18		23.66	8.70	
**Epo dose/kg** (U/kg)	HDF	−3.57	2.11	**0.04**	−11.85	10.32	**0.01**
	HD	−2.18	1.54		6.47	26.48	
	Once	−3.58	2.18	0.9	−10.83	9.15	0.8
	Twice	−3.57	2.15		−12.76	11.69	
**(EPO/kg) /Hct Ratio**	HDF	−10.42	11.42	**0.03**	−24.14	16.50	**0.00**
	HD	0.36	12.79		7.92	36.19	
	Once	−6.41	11.35	0.1	−20.68	20.53	0.6
	Twice	−13.75	10.81		−27.25	12.13	
**Iron Dose** (mg/month)	HDF	−5.12	27.86	0.5	−21.76	25.98	**0.03**
	HD	−0.03	30.23		−0.97	31.62	
	Once	−12.9	33.26	0.3	−22.26	33.09	0.6
	Twice	1.88	21.3		−21.30	19.41	
**Dialysis costs** (EGP/month) (US$/month)	HDF	42.5 (5.667)	14.27 (1.903)	**0.00**	42.58 (5.677)	14.29 (1.905)	**0.00**
	HD	0.02 (0.003)	0.25 (0.033)		0.02 (0.003)	0.318 (0.0424)	
	Once	27.92 (3.723)	0.29 (0.039)	**0.00**	27.93 (3.724)	0.391 (0.052)	**0.00**
	Twice	55.74 (7.432)	0.35 (0.0467)		55.77 (7.436)	0.54 (0.072)	
**Non-Dialysis costs** (EGP/month) (US$/month)	HDF	−5.72 (−0.763)	0.96 (0.128)	**0.00**	−9.65 (−1.287)	8.93 (1.191)	**0.00**
	HD	−0.56 (− 0.075)	1.88 (0.251)		93.68 (12.491)	198 (26.4)	
	Once	−4.56 (−0.608)	1.84 (0.245)	**0.00**	−7.11 (−0.948)	7.05 (0.94)	**0.02**
	Twice	−6.7 (−0.893)	1.46 (0.195)		−11.93 (−1.591)	10.16 (1.355)	
**Net costs/m** (EGP/month) (US$/month)	HDF	21.45 (2.86)	7.20 (0.96)	**0.00**	19.81 (2.6410	7.32 (0.976)	**0.00**
	HD	−0.08 (−0.011)	0.70 (0.093)		9.47 (1.263)	17.36 (2.315)	
	Once	14.22 (1.896)	1.50 (0.2)	**0.00**	13.27 (1.769)	2.12 (0.283)	**0.00**
	Twice	27.96 (3.728)	1.53 (0.204)		25.69 (3.425)	4.69 (0.625)	

*EPO* Erythropoietin, *Hct* hematocrit value

**Fig. 5:**
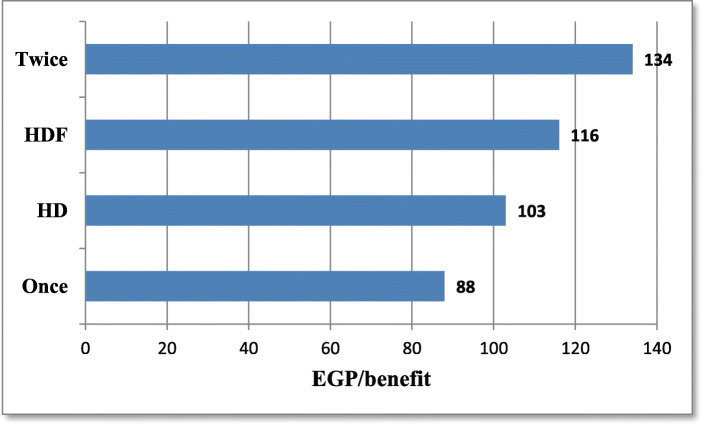
Bar chart for cost benefit ratio in different groups. *To compare cost/benefit ratio between the different groups and subgroups, calculate the incremental costs mean and divide it by the total benefit score mean, as the following: Incremental costs mean /benefit mean in HDF group = 554/4.78 = 116(EGP/benefit). Incremental costs mean /benefit mean in HD group = 207 /2 = 103(EGP/benefit). Incremental costs mean /benefit mean in once subgroup = 362.4/4.11 = 88 (EGP/benefit). Incremental costs mean /benefit mean in twice subgroup = 727.8/5.4 = 134(EGP/benefit)*

-Comparisons between different patients’ groups as regards the benefit score and cost benefit ratio are shown in Table 7, 8, Fig. 5. All patients on HDF have a positive total benefit score in contrast to patients on HD who have only 33% with positive total benefit score.

**Table 7:** Comparison between different patients’ groups as regards the benefit score category

	Benefit Score Category				χ^**2**^	p
	Benefit (+ve total score)	No change (0 total score)	Deterioration (−ve total score)	Total		
HDF	19 (100%)	0	0	19	17	**0.00**
HD	4 (33%)	2 (16.7%)	6 (50%)	12		
Total	23	2	6	31		
Once	9 (100%)	0	0	9	0	1
Twice	10 (100%)	0	0	10		
Total	19	0	0	19

**Table 8:** Comparison between different patients’ groups as regards the cost benefit ratio

Variable	Group	Mean	Median	±SD	IQR	Z	p
**Cost/benefit** **Ratio** (EGP/benefit)	HDF	124.51	110.85	56.81	74	1.54	0.1
	HD	196.83	14	372.82	566		
	Once	96.64	78.8	38.9	37.68	2.28	**0.02**
	Twice	149.59	132	60.28	88.4		
	Once versus HD					1.38	0.1
	Twice versus HD					1.4	0.1

## Discussion

The present study was a sequential (2 phases) clinical follow up study and was aiming at evaluation of the benefits versus costs of substitution of once or twice weekly HD sessions with OL-HDF ones. The purpose of the initial phase was to exclude the occurrence of spontaneous improvement in the studied patient groups before introducing the OL-HDF sessions.

The study showed that the substitution of HD sessions with OL-HDF sessions improved growth compared to pure HD sessions. This is demonstrated by the percent change of weight and height SDS which were significantly higher in HDF group than the HD group, both on short term and long term. Furthermore, once weekly HDF subgroup and twice subgroup had equivalent effect on growth (weight and height), as there was no statistically significant difference between them on either short term and long term evaluation. The improvement in growth which occurred in the present study could be explained by the better dialysis adequacy, clearance of middle molecules and correction of chronic inflammatory state.

***Fischbach*et al.*, (2010)*** demonstrated that daily OL-HDF promotes catch-up growth in children on chronic dialysis [22]. However, children in our study were still stunted compared to normal population (no catch up, SDS for weight for age and height for age in HDF group at the end of the study were − 4.4 and − 3.5 respectively). That is the difference between our study and their study because they used “daily” schedule while we used a more “economic” approach. The 3H study is set to address the effect of HDF on growth and cardiovascular system [23]. Molina et al., 2018 compared HDF with high-flux HD. HDF was associated with preservation of muscle mass, increased protein intake, and reduced inflammation, suggesting that HDF could help prevent protein-energy wasting and promote growth [24].

In our study, there were no significant differences between different groups as regards blood pressure categories. But there was some improvement in its control reflected by shift of some patients from uncontrolled category to controlled category. Many authors also support the evidence that there is no significant change in blood pressure values between the convective (HF &OL-HDF) and the diffusive (LF-HD) therapies [14, 25–29].

The HDF group showed decreased frequency of intradialytic symptomatic hypotension (ISH) compared to HD group, yet not reaching a significant difference. This is demonstrated by the decrease of percent change in ISH frequency means in HDF group and increase in the HD group. Also, the twice subgroup showed better improvement in ISH frequency with no statistically significant difference compared to once subgroup both on short term and long term. This came in agreement with many studies that showed a lower ISH frequency in patients who were treated with HDF and HF compared to patients who were treated with low-flux HD, [5, 30–35] others disagree [36, 37].

Fatigue is one of the most common symptoms in hemodialysis patients with a prevalence of 65% [38]. In the present study, the HDF group showed significant decrease in percent change of post-dialysis fatigue frequency compared to HD group (which actually increased in the latter), both on short term and long term. Also twice subgroup showed statistically significant decrease (negative percent change) of frequency of post-dialysis fatigue on short term only. However, this difference became insignificant on long term suggesting that once weekly HDF might have a slower effect. It is common in clinical practice to find that patients with inadequate dialysis suffer fatigue [39]. This comes in agreement with some studies that reported a statistically significant association between Kt/V and fatigue [40]. Several factors may be implicated e.g. anemia, malnutrition and chronic inflammation [39]. Cytokines may cause fatigue either through activation of the central nervous system, hypothalamus, pituitary gland, and adrenal glands or by inducing sleep disorders, depression, or anxiety [41]. Moreover a significant association between fatigue and serum IL-6 has also been demonstrated in HD patients [42].

In this study, the HDF group showed improvement of anemia compared to HD group. This is demonstrated by the significant increase in hemoglobin levels, and decrease in iron dose, Epo dose/kg and Epo /Hct ratio (a marker of erythropoietin resistance). These changes occurred both on short term and long term. Also, twice subgroup demonstrated a significant increase in hemoglobin level on short term only, to become non-significant on long term. This could be due to the assumed slower effect of once weekly HDF and that improvement of anemia occurred with introduction of HDF regardless of the frequency. The pathogenesis of renal anemia is multifactorial; presence of erythropoietin inhibitors, inadequate dialysis and low Kt/V, hyperparathyroidism [43] and chronic inflammation [44, 45]. From our results, we suggested that OL-HDF has corrected the above pathogenic factors, also the use of high quality water that can reduce inflammation is thought to share in this correction so the anemia was improved [46, 47]. This came in agreement with many studies [46–51].

***Stefansson*et al*(2012***)found that OL-HDF decreased serum levels of hepcidin (HEP) which is a major pathogenic factor in renal anemia [49]. A recent randomized trial investigating erythropoietin resistance index (ERI) (weekly Epo dose/ kg/ gmHb) in dialysis patients, demonstrated that OL- HDF caused significant reduction of ERI values [47] However, CONTRAST study found no effect on erythropoiesis-stimulating agent resistance [52] and a meta-analysis confirmed the finding [53].

The present study showed that OL-HDF –compared to HD- improved serum Ca levels, yet not reaching a significant difference. Some studies demonstrated that HDF decreases serum ionized Ca by increasing its clearance [54, 55]. However, our patients were slightly hypocalcemic at the start of the study (mean 7.7–8.1) and we assume that increased serum Ca was due to the improved nutrition. Moreover, OL-HDF improves response to vitamin D action, so it increases gastrointestinal Ca absorption [56].

Our results showed a significant difference in percent change in the predialysis serum P in between HDF and HD groups, being decreased (negative) in the former and increased (positive) in the latter. This significant difference was on short term only and became non-significant on long term. The phosphorus decrease is due to better clearance by convective force used in HDF [10, 57]. OL-HDF proved to decrease serum levels of fibroblast growth factor-23(FGF-23) decreasing its harmful effects on calcium phosphate metabolism [58]. Contradictory results were stated by other studies; one study found the amount of phosphate removed with HDF was 15–20% greater than that with high-flux HD [59], whereas in the second study, no difference was found [60]. Furthermore, in the CONTRAST Study, predialysis serum phosphate levels were reduced by 6%, and the percentage of patients reaching target pretreatment serum phosphorus levels increased from 64 to 74% [10]., ***Francisco*et al.*(2013)*** stated that OL-HDF was associated with better control of serum phosphorus fraction, compared with hemodialysis [61]. The loss of significance on long term and also the conflicting results of once and twice subgroups could be explained by the fact that serum P is not dependent only on dialysis modality but it depends more on dietary restriction and compliance to phosphorus binders which may vary and could have affected our results.

In the present study, OL-HDF group showed a significant difference in percent change of PTH levels both on short term and long term. In addition, both once and twice subgroups showed decrease in PTH with significant difference between them on short term but not on long term. In agreement, ***Wang*et al.*(2004)*** proved that HDF clears PTH but HD did not [62]. ***Movilli*et al.*(2011)*** demonstrated that after 6 months on OL-HDF, P and PTH levels significantly decreased compared to the pre HDF levels. This could be explained also by the rise in serum Ca and the decrease in serum P mentioned above [63].

The present work proved an enhanced β_2_m clearance by HDF where a highly significant decrease in β_2_m was revealed on long term only. Furthermore the twice subgroup showed more decrease in predialysis β_2_m than once subgroup but not reaching a significant difference. This beneficial effect probably results from use of ultrapure fluids and biocompatible materials reducing inflammation combined with convective therapy that enhances β_2_m removal and this is a well-established fact proved in many studies [1, 57, 62, 64–67]. Again, different modalities of implicating HDF proved beneficial.

In ESRD, concentrations of both pro- and anti-inflammatory cytokines are several folds higher, probably due to decreased renal clearance and increased production [68]. To assess the effect of HDF on inflammatory state we studied IL-6 and hs-CRP. Our results revealed that the OL-HDF caused highly significant decrease in both parameters, both on short term and long term. Also the twice group showed a more significant decrease than once group, while the HD group actually showed increase in levels of these parameters. Our results came in agreement with another Egyptian study [69]. Also, many studies demonstrated control of the inflammatory state on OL-HDF [64, 70–72]. On the contrary, some other studies reported that CRP or IL-6 levels remained stable [11, 57, 73–75]. Moreover, ***den Hoedt*et al.*(2014)*** analyzed data from CONTRAST study and found that HD has increased the levels of IL-6 and CRP while their levels remained stable in patients treated with OL-HDF [76].

Our results proved that HDF significantly increased weekly Kt/V compared to HD. Also, twice subgroup significantly increased weekly Kt/V compared to once subgroup. This is explained by the higher efficiency of dialysis [47, 63] others don’t agree [29, 62].

The HDF group showed significant increase in dialysis costs compared to HD group. Same was found in twice subgroup compared to once subgroup, both on short term and long term. The interesting point in our results is that the non-dialysis costs in HDF group significantly decreased compared to HD group (which actually increased). This is due to the decrease in costs of the drugs (especially, ESAs, phosphate binders and activated Vitamin D). So when we compared the net costs increase between HDF group and HD group at the end of the study, it was found to be statistically insignificant difference.

When studying cost effectiveness of different modes of dialysis in adults, the effect of dialysis is measured by combined measurement of both quantity (survival or mortality rate) and quality of life (QoL) and merging these two parameters into single variable (Quality Adjusted Life or QALY). This can be used statistically to calculate the cost effectiveness as costs/QALY gained [77].

In Egypt, there is no translated questionnaire for assessment of QoL in pediatric ESRD patients and we have no mortality in our study. There is no regional registry collecting data on ESRD and its outcome [78]. Also, we have no defined Society’s willing to pay (WTP) in Egypt.

So, in our study, we created the idea of benefit score because we could not calculate the QALY gain. The total benefit score was significantly different between HDF group and HD group. We demonstrated that all HDF group (100%) had a positive total benefit score while only 33% of HD group had a positive total score and 50% of HD patients have a negative total benefit score. The cost/benefit ratio in HDF group was not statistically different from HD group. We also demonstrated that the once subgroup has the least cost benefit ratio, while the twice subgroup has the highest cost benefit ratio.

***Mazairac*et al.*, (2013)*** performed a detailed cost comparison of HD and HDF in adults on data obtained from CONTRAST study. They found that HDF was slightly more expensive than HD. Although the difference between the two modalities was small, it was not outweighed by the limited QALY gain, so they concluded that HDF is not cost-effective compared to HD [15]. However, another study stated that adequate water treatment and the use of ultrapure dialysate have a favorable cost benefit for dialysis units [79].

The present study, as the first study addressing cost benefit relationship of HDF and HD in children, showed that incremental cost for HDF is acceptable if compared to its clinical and laboratory benefits. The strength of our study comes from the randomized controlled design and the double phase design. Also, to our knowledge, this is the first study to target the cost benefit relationship of HDF and HD in children.

There are some limitations of our study. Firstly, the period of following up the patients on HDF was relatively short (only 15 months). Secondly, the small number of patients included but still it’s a reasonable number compared to smaller pediatric ESRD community. Thirdly, not all the costs were documented in details. Lastly, we could not calculate the QALY gain as mentioned above.

**In Conclusion** The substitution of once or twice weekly HD sessions with OL-HDF sessions was beneficial in most of the clinical and laboratory parameters measured. On long term, the twice weekly HDF sessions were statistically better than once in correction of inflammatory profile, elevation of Kt/V and more reduction in non-dialysis costs. Otherwise, no statistically significant difference was found between once and twice weekly HDF. The nearly equivalent clinical results in once and twice HDF groups can be due to the limited follow up period. Once weekly HDF had the least cost benefit ratio. So, the way we see it, once weekly HDF is the most cost-effective OL-HDF and may have very promising results in children with ESRD for whom transplantation is not always feasible (such as in our society). The alternative regimens we suggested (once weekly and twice weekly HDF) can represent a breakthrough in renal replacement therapy in children with ESRD in developing countries.

The authors would like to mention that cardiovascular benefit of hemodiafiltration was not tackled in their work neither through measuring plasma indicators such as serum L-carnitine nor through performing echocardiography. Further studies concerning this subject are planned.

## Data Availability

All data enrolled in the study are available within their records at our unit. The corresponding author is the one to be contacted in case any data is needed.

## Abbreviations

HD: Hemodialysis
ESRD: End stage renal disease
HDF: Hemodiafilteration
OL-HDF: Online hemodiafilteration
RRF: Residual renal function
ICER: Incremental cost effectiveness ratio
Q_b_: Blood flow rate
Q_d_: Dialysate flow rate
ISH: Intradialytic symptomatic hypotension
Epo: Erythropoietin
Wt: Weight
Ht: Height
SDS: Standard Deviation Score
Bp: Blood pressure
Ca: Calcium
PO4: Phosphorus
PTH: Parathyroid hormone
β_2_m: β_2_ microglobulin
FGF-23: Fibroblast growth factor-23
IL-6: Interleukin 6
CRP: C- reactive protein
CBC: Complete blood count
Hb: Hemoglobin
HCT: Hematocrit
OCM: Online conductivity monitoring
EGP: Egyptian pound
ESA: Erythropoietin Simulating Agents
TMP: Trans-membrane pressure
hs-CRP: Highly sensitive C- reactive protein
LF-HD: low flux hemodialysis
HEP: Hepcidin
ERI: Erythropoietin resistance index
QoL: Quality of life
QALY: Quality Adjusted Life Year
WTP: Society’s willing to pay
